# Supplementary material for: A Randomized Hybrid Type I Effectiveness-Implementation Study of an eHealth Delivery Alternative for Cancer Genetic Testing for Hereditary Cancer (eREACH2): study protocol
Source: BMC Cancer. 2026 Jul 31;26:1307. doi: 10.1186/s12885-026-16620-z (PMC13711929; doi:10.1186/s12885-026-16620-z)
Supplement: Supplementary file 3 [file 12885_2026_16620_MOESM2_ESM.docx]

| **Supplementary Table 2:** Summary of key changes made during development and design of the digital intervention based on user comments during user and usability testing. | |
| --- | --- |
| **Key changes recommended** | **Representative user comments** |
| User testing |  |
| -simplify content | -a lot of information, maybe use more bullets (V1)  -Keep it simple, especially if someone already has it--getting to technical here- could lose a lot of people-- want more focus how it can help you (V1)  -might be a little too much, possibly split into 2 slides (V2) |
| -address confusing content | -word somatic confuses initially, nice to be able to get defintions of words when you hover over (easier and faster than glossary)(V1)  -switch between inherited and germline confusing- make the order consistent (V1)  -what family members? Children, siblings, parents or distant? (V2) |
| -address benefits of testing equally to risks and limitations | -Too much info on uncertainty. Will just make people nervous (V1) |
| -add more information on costs and consider addressing earlier | -This info needs to be clear earlier. Could be a big barrier. Maybe something about speaking to a gc with concerns (V1)  -0-100 I can afford, should be higher up in the presentation, it is important to know (V1) |
| -move content to Tier 2 or Tier 1 | -[example case scenario] doesn't mean anything to me at this point- not useful or helpful- should be extra category (V1)  -needs to be in the extra category- might make people's eyes glaze over (V1)  -reinforces decision making – thinks this should be in tier 1 (V1) |
| Usability testing |  |
| -clearer instructions and labeling | -unclear how to advance through a set of 4 screens – make arrows more clear (V1)  -have a back arrow in case someone changes their mind and doesn’t want to proceed to results (V2)  -placement of the help button is confusing (V2) |
| -functionality | -videos are slow (V1)  -page did not load (V2) |
| -changes in size of text and figures | -diagram is too small to read (V1)  -text is too small or should be bold (V2) |
| -changes to images | -photo of physician is intimidating (V1)  -need more photos of older people (V2) |
